# The impact of the COVID-19 pandemic on resuscitation attempts, bystander CPR and survival outcomes in Australia and New Zealand: A binational population-based, Epistry study

**DOI:** 10.1016/j.resplu.2025.100894

**Published:** 2025-02-07

**Authors:** Stuart Howell, Ziad Nehme, Stephen Ball, Tan Doan, Judith Finn, Emma Bosley, Steven Faddy, Bridget Dicker, Andy Swain, Peter Cameron, Melanie Thorrowgood, Andrew Thomas, Samuel Perillo, Mike McDermott, Matt Green, Nicole Packham, Ashanti Dantanarayana, Joe Cuthbertson, Janet Bray, Aus-ROC OHCA Epistry Management Committee

**Affiliations:** a School of Public Health and Preventive Medicine, Monash University, Victoria, Australia; b Department of Paramedicine, Monash University, Victoria, Australia; c Ambulance Victoria, Victoria, Australia; d Prehospital, Resuscitation and Emergency Care Research Unit (PRECRU), Curtin University, Western Australia, Australia; e St John Western Australia, Western Australia, Australia; f Queensland Ambulance Service, Queensland, Australia; g School of Medicine (Emergency Medicine), University of Western Australia, Australia; h School of Clinical Sciences, Queensland University of Technology, Queensland, Australia; i NSW Ambulance, New South Wales, Australia; j Hato Hone St John New Zealand, Auckland, New Zealand; k Auckland University of Technology, Auckland, New Zealand; l Wellington Free Ambulance, Wellington, New Zealand; m Emergency and Trauma Centre, The Alfred, Melbourne, Victoria, Australia; n SA Ambulance Service, South Australia, Australia; o St John Ambulance NT, Northern Territory, Australia; p ACT Ambulance, Australian Capital Territory, Australia; q Ambulance Tasmania, Tasmania, Australia

**Keywords:** Out-of-hospital cardiac arrest, Registry, Emergency medical services, COVID-19

## Abstract

**Aim:**

This study aims to assess the impact of the COVID-19 pandemic on out-of-hospital cardiac arrest (OHCA) incidence, bystander cardiopulmonary resuscitation (CPR), EMS resuscitation attempts and survival across Australia and New Zealand.

**Method:**

Data were extracted for all OHCAs patients attended by emergency medical services (EMS) between 2017 and 2021 from the Aus-ROC OHCA Epistry (Epidemiological registry). Logistic regression was used to explore differences between the pre-COVID-19 (January 1, 2017 to March 15, 2020) and COVID-19 (March 16, 2020 to December 31, 2021) periods for bystander CPR, EMS-attempted resuscitation, survival to hospital arrival (event survival) and survival to hospital discharge/30 days.

**Results:**

The incidence of OHCA increased during COVID-19 in Australia and New Zealand, although this varied regionally. When compared to the pre-COVID-19 period, COVID-19 was associated with a significant increase in the odds of an EMS-attempted resuscitation in Queensland (adjusted odds ratio (aOR) = 1.19; 95%CI: 1.01–1.40, p = 0.03) and Western Australia (aOR = 1.26; 95%CI: 1.03–1.54, p = 0.02). The COVID-19 period was associated with a decrease in survival to hospital arrival in Australia overall (aOR = 0.91; 95% CI:0.83–0.99, p = 0.04), and by region in Victoria (aOR = 0.74; 95% CI:0.63–0.87, p < 0.01) and Tasmania (aOR = 0.48; 95% CI:0.25–0.91, p = 0.02), and with a decrease in survival to hospital discharge/30 days in Australia (aOR = 0.82; 95% CI:0.70–0.96, p = 0.01), and by region in Victoria (aOR = 0.70; 95% CI:0.54–0.91, p < 0.01) and South Australia (aOR = 0.61; 95% CI:0.37–0.99, p = 0.04). There were no significant changes in survival during COVID-19 in New Zealand.

**Conclusion:**

Regional variations were observed with respect to the associations of COVID-19 with resuscitation attempts and OHCA survival.

## Introduction

COVID-19 first emerged in December 2019 and was declared a global pandemic by the World Health Organisation (WHO) in March 2020. In Australia, the federal government declared a state of emergency on March 16th, and by December 2021, there were 395,504 confirmed cases and 2,239 deaths (Supplementary file^,^ Table S1). During the same period, there were 13,852 cases and 54 deaths in New Zealand. Australia and New Zealand had fewer cases and deaths per million people than many countries including North America and Europe.^1^ When compared to 38 OECD (Organization for Economic Cooperation and Development) countries, Australia and New Zealand had the smallest outbreaks with respect to size and spread.^2^

In international studies, COVID-19 has been linked to an increased incidence of out-of-hospital cardiac arrest (OHCA) and to lower rates of survival to hospital admission and survival to hospital discharge/30 days.^3^, ^4^, ^5^, ^6^, ^7^, ^8^ The drivers of increased OHCA incidence and mortality are likely to be multifaceted, however, pandemic-related changes in pre-hospital factors such as arrest rhythm, aetiology, location and emergency medical service (EMS) response times are likely to be important.^3^ Differences across nations and regions have been reported with respect to pandemic-related changes in OHCA incidence and survival, which may reflect differences in COVID-19 caseload, local public health responses to the pandemic, as well as demography, surveillance and data collection processes.^6^

The impact of COVID-19 on OHCA incidence and outcomes in Australia and New Zealand remains to be fully elucidated. Two Australian studies from the states of Victoria and Western Australia (WA) suggest that COVID-19 had a minimal impact on OHCA incidence in our region,^9^, ^10^ but was associated with poorer survival outcomes in Victoria. However, both studies restricted their investigation to the first wave of the pandemic (March to May 2020), and the longer-term impact of COVID-19 on OHCA in our region remains to be explored. The purpose of this study is to examine OHCA incidence, bystander cardiopulmonary (CPR) rates, EMS resuscitation attempts and survival during the first two years of the COVID-19 pandemic compared to the pre-pandemic period across Australia and New Zealand.

## Method

### Study setting and population

This retrospective, population-based study of OHCA across Australia and New Zealand used data from the Australasian Resuscitation Outcomes Consortium (Aus-ROC) Australian and New Zealand OHCA Epistry (Epidemiological Registry) for the years 2017 to 2021. The study population included all OHCA attended by emergency medical services (EMS-attended OHCA), with EMS-witnessed arrests excluded from the outcome analysis.

### Study period

In this study, the pre-COVID-19 period is defined as January 1st 2017 to March 15th 2020 and the COVID-19 period from March 16th 2020 to December 31st 2021. COVID-19 case numbers for this period are shown in the Supplementary file (Table S1). The incidence of COVID-19 was higher in Australia (859.4 cases per 100,000 person years) than in New Zealand (155.3 cases per 100,000 person years) (Table 1). COVID-19 incidence varied across regions, ranging from 23.4 cases per 100,000 person years in Western Australia to 1487.7 cases per 100,000 person years in Victoria.

**Table 1:** Incidence of locally-acquired COVID-19 infections in Australia and confirmed cases in New Zealand 2020–2021 (cases per 100,000 population).

	March 16th 2020-31st December 2021 Crude incidence of locally-acquired COVID-19 infections per 100,000 person-years (n infections)^1^	Pre-COVID-19 (January 1st 2017-15th March 2020) Crude incidence of OHCA per 100,000 person-years (95% CI)^2^	COVID-19 (March 16th 2020-31st December 2021 Crude incidence of OHCA per 100,000 person-years (95% CI)^3^
Australia	859.4 (395,207)	105.5 (104.8–106.3)	111.0 (110.1–112.0)
Victoria	1487.7 (176,477)	100.1 (98.7–101.5)	105.9 (104.0–107.7)
South Australia	344.4 (11,058)	122.6 (119.7–125.5)	122.8 (119.0–126.7
Western Australia	23.4 (1,140)	99.4 (97.3–101.5)	103.8 (101.0–106.7)
Queensland	149.0 (13,802)	108.1 (106.5–109.7)	113.5 (111.3–115.7)
Northern Territory	128.9 (572)	*	*
Tasmania	77.9 (779)	127.6 (122.4–133.1)	111.5 (105.2–118.2)
Australian Capital Territory	502.5 (4,009)	*	*
New South Wales	1288.2 (187,370)	105.1 (103.9–106.4)	113.5 (111.7–115.2)
New Zealand	155.3 (14,174)	108.2 (106.6–109.9)	113.7 (111.5–115.9)
New Zealand^4^	na	112.0 (110.2–113.7)	114.6 (112.3–116.9)
Wellington region	na	77.1 (73.0–81.4)	106.1 (99.8–112.8)

na: Not available.

1 Estimated resident populations as at June 2020.

2 Estimated resident populations as at June 2018.

3 Estimated resident population as at June 2020.

4 New Zealand excluding Wellington region.

* Australian Capital Territory and Northern Territory only provide cases for EMS treated arrests.

Responses at the country and regional level were targeted to limit community spread of COVID-19. Similar strategies were adopted across the region and included movement restrictions (state border closures, lockdowns and work-from-home directives), mask mandates and social distancing, testing and contact tracing and quarantine periods for those exposed to the virus and vaccination. However, Victoria and New South Wales (NSW) had longer periods of lockdown than other regions due to a greater number of periods of increased levels of transmission through the community. Since the public health response to COVID-19 varied somewhat across our region, data is presented for Australia and New Zealand, as well as each of the 10 regions serviced by the EMS.

### Data source

The data were sourced from the Aus-ROC OHCA Epistry which collects data from the regional OHCA registries of all ambulance services in Australia and New Zealand. In Australia, each of the eight ambulance services cover an individual state or territory, while in New Zealand, one ambulance service (Wellington Free Ambulance) covers the Greater Wellington region and the other (Hato Hone St John New Zealand) covers the rest of the country. In combination, the 10 ambulance services provide complete coverage across the two nations, encompassing a land area of 7.96 million km^2^ and a population of approximately 30 million people.^11^

OHCA is defined as the cessation of cardiac mechanical activity occurring outside of the hospital setting, confirmed by the absence of signs of circulation. All OHCA attended by EMS are included in the Epistry regardless of aetiology. Obvious deaths attended by EMS are classified as OHCA and are included in the Epistry, as are patients who received a successful attempt at defibrillation prior to EMS arrival.^10^

With the exception of two Australian regions, each participating ambulance service provides data for all OHCA patients attended by EMS. The ambulance services of the Northern Territory and Australian Capital Territory only provide data for patients for whom EMS attempted resuscitation. These services provide a small number of cases (n = 300 cases/year) and are excluded from the analysis of incidence. The Aus-ROC Epistry data are collected according to the 2015 Utstein definitions^12^ and are subject to quality control. Cases are identified from the patient report forms by the individual EMS, who conduct data validation according to their own quality control procedures. The data are then forwarded to the Aus-ROC Epistry where further integrity checks are completed.

A detailed description of the Epistry can be found elsewhere.^11^, ^13^

### Study variables

***Outcomes:*** Differences between the pre-COVID-19 and COVID-19 periods are described and compared for OHCA incidence, bystander CPR, whether an EMS resuscitation attempt was made, event survival (return of spontaneous circulation (ROSC) at hospital handover), and survival to hospital discharge/30 days. EMS attempted resuscitation was defined according to the latest (2024) Utstein template,^14^ as cardiopulmonary resuscitation (CPR) and/or defibrillation provided by the attending EMS paramedic, or received bystander defibrillation with ROSC prior to EMS arrival.^14^ Bystander CPR, event survival (return of spontaneous circulation (ROSC) at hospital arrival) and survival to hospital discharge/30 days were assessed in the subset of OHCA patients where a resuscitation attempt had been made. In our data, some EMS report survival to hospital discharge, while others report 30-day survival. Survival to hospital discharge has been shown to be equivalent to 30-day survival as a survival outcome for OHCA.^15^ Survival to hospital discharge/30 days was not available for Queensland, the Northern Territory or the Australian Capital Territory. For the purposes of bystander CPR, a bystander is defined as any individual on scene who is not part of the dispatched first responder team.

In this study, “unknown” responses were treated as missing values and excluded from the analysis. The percentage of unknown responses was low for most variables (Supplementary file^,^ Table S2), and occurred predominantly amongst cases that had died at scene and did not receive a resuscitation attempt.

### Statistical analysis

Crude incidence rates are reported as the number OHCA cases per 100,000 person years. Estimated residential populations (ERP), as sourced from the Australian Bureau of Statistics^16^ and Statistics New Zealand, were used to calculate the incidence for the pre-COVID-19 period (ERP at June 2018) and COVID-19 period (ERP at June 2020) (Supplementary file^,^ Table S3).

Data concerning the impact of COVID-19 on the outcome measures are presented as crude odds ratios (OR) and adjusted odds ratios (aOR) with 95% confidence intervals as obtained using logistic regression. Odds ratios for EMS attempted resuscitation and survival outcomes were adjusted for patient age and sex, arrest location, arrest aetiology, initial arrest rhythm, witnessed status, bystander CPR, EMS response times and season. These factors are known to influence OHCA survival in our region^17^ and are likely to be associated with EMS resuscitation attempts. Odds ratios for bystander CPR were adjusted for all of the above factors, with the exception of initial arrest rhythm as arrest rhythm is unlikely to influence the provision of CPR from a bystander’s perspective. Both the crude and adjusted odds ratios were adjusted for linear trend using yearly quarter as a time variable to ensure that our results reflected the impact of COVID-19 independently of any underlying trend. Defibrillation prior to EMS arrival was not included as a covariate in the analysis as it formed part of the case definition of treated OHCA and caused issues with collinearity and perfect prediction in some models. Issues were identified from error messages and warnings on the computer output; we also screened for unusually large or small standard errors indicative of problems with collinearity.

Cases with missing data on one or more of the variables of interest were excluded from the analysis. The analyses were completed using Stata Version 17.0 (StataCorp, College Station, TX, USA).

## Results

**Fig. 1:**
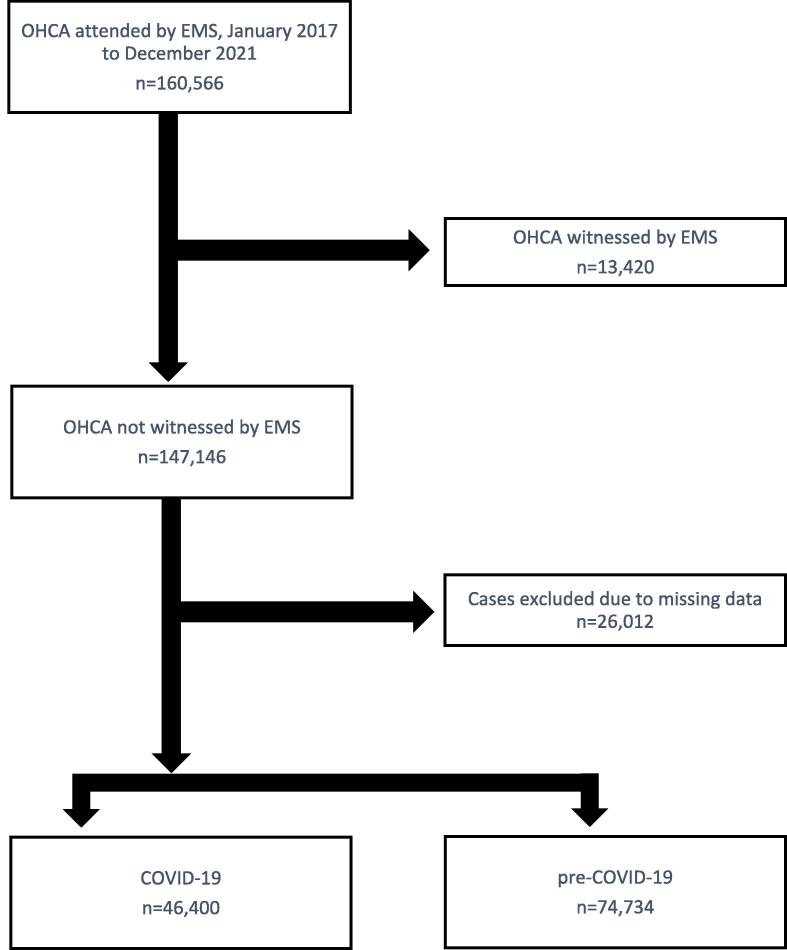
Study flow diagram for all OHCA attended by EMS.

**Fig. 2:**
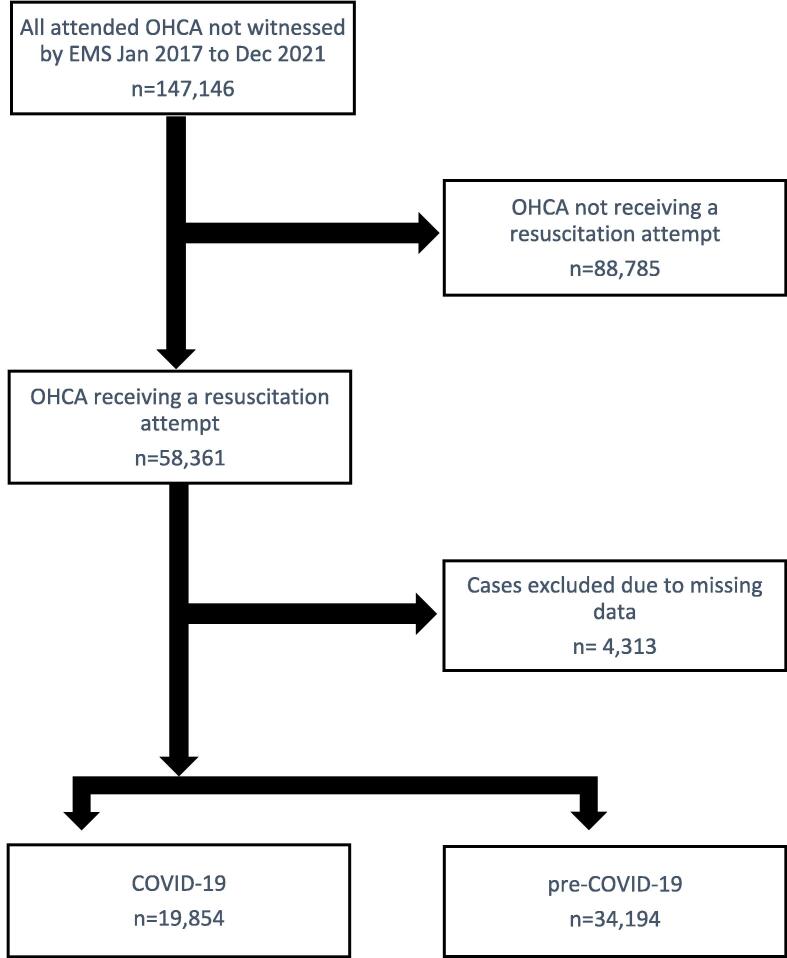
Study flow diagram for OHCA receiving a resuscitation attempt.

There were 160,566 OHCA patients attended by EMS in Australia and New Zealand between January 2017 and December 2021. When EMS-witnessed cases were excluded (n = 13,420), there were 147,146 OHCA patients attended by EMS. Following further exclusion of cases with missing data, there were 74,734 cases during the pre-COVID-19 period and 46,400 during COVID-19 (Fig. 1). There were 58,361 OHCA patients who received an attempted resuscitation by EMS. After exclusions due to missing data, there were 34,194 cases during the pre-COVID period and 19,854 cases during COVID-19 (Fig. 2).

The overall incidence of OHCAs, including EMS-witnessed arrests was higher in the COVID-19 period than the pre-COVID-19 period in both Australia and New Zealand (Table 1). This was observed in all regions except South Australia where there was no significant change in OHCA incidence across the study periods, and Tasmania where OHCA incidence was lower during COVID-19.

### Characteristics of OHCA patients attended by EMS: pre-COVID-19 versus COVID-19 periods

When compared to the pre-COVID-19 period, the COVID-19 period was associated with an increase in the proportion of cases that arrested in a private residence (77.7% vs 80.6%) (Table 2). There was an increase in the proportion of arrests of medical aetiology (84.2% vs 85.1%), a decrease in the proportion of arrests with an initial shockable rhythm (13.5% vs 11.4%) and a decrease in the proportion of arrests where resuscitation was attempted (45.4% vs 42.5%) (Table 2). Median EMS response times were longer during COVID-19 (8.1 vs 9.2 min), and patients were slightly older (67 vs 68 years) (Table 2). There was a small increase in automated external defibrillator device (AED) use (2.0% vs 2.4%) but no change in the proportion of cases receiving bystander CPR (41.8% vs 41.8%) (Table 2). Unadjusted survival outcomes for EMS attended OHCA patients were worse during COVID-19 (event survival: 11.4% vs 9.1%; survival to discharge/30 days: (4.9% vs 3.6%) (Table 2).

**Table 2:** Demographic characteristics, cardiac arrest features and on-scene intervention for all attended OHCA not witnessed by EMS during the pre-COVID-19 and COVID-19 periods.

	Pre-COVID-19 1st January 2017-15th March 2020 N = 74,734	COVID-19 16th March 2020-31st December 2021 N = 46,400
Attempted resuscitation, % (n)	45.4 (33,742)	42.5 (19,595)
Age, median (IQR)	67 (52, 80)	68 (53,80)
Male, % (n)	66.4 (49,292)	66.2 (30,516)
Location of arrest, % (n)		
Private residence	77.7 (57,721)	80.6 (37,139)
Public place	13.7 (10,159)	11.5 (5,309)
Other	8.6 (6,391)	7.9 (3,649)
Bystander witnessed arrest, % (n)	31.4 (23,298)	32.2 (14,852)
Aetiology, % (n)		
Medical	84.2 (62,516)	85.1 (39,241)
Non-medical	15.8 (11,755)	14.9 (6,856)
Arrest rhythm, % (n)		
Shockable	13.5 (10,028)	11.4 (5,236)
Non-shockable	86.5 (64,243)	88.6 (40,861)
Bystander CPR, % (n)	41.8 (31,083)	41.8 (19,284)
Shocked by AED, % (n)	2.0 (1,485)	2.4 (1,129)
EMS response time (minutes), median (IQR)	8.1 (6.0, 12.0)	9.2 (6.9, 14.0)
Scene outcome, % (n)		
Died at scene	82.5 (61,305)	86.3 (39,779)
Transported	17.5 (12,962)	13.7 (6,312)
Survived event, % (n)	11.4 (7,397)	9.1 (3,662)
Survived to discharge/30 days, % (n)	4.9 (3,151)	3.6 (1,457)

### Characteristics of OHCA patients receiving a resuscitation attempt: pre-COVID-19 versus COVID-19 periods

There were 54,048 OHCA patients that received a resuscitation attempt over the study period (34,194 and 19,854 OHCA during the pre-COVID-19 and COVID-19 periods, respectively) (Table 3). When compared to the pre-COVID-19 period, the COVID-19 period was associated with an increase in the proportion of arrests in private residences (71.3% vs 75.3%) (Table 3). The proportion of OHCA patients that died on scene was higher during COVID-19 (62.1% vs 68.3%), and median EMS response times were longer (8.0 vs 9.0 min) (Table 3). The proportion of OHCA patients presenting with an initial shockable rhythm (29.2% vs 26.0%) was lower during COVID-19, while AED use (4.3% vs 5.4%) and bystander CPR (74.7% vs 76.8%) were higher (Table 3). Unadjusted event survival (25.8% vs 22.5%) and survival to hospital discharge/30 days (11.1% vs 8.9%) were lower during the COVID-19 period (Table 3).

**Table 3:** Demographic characteristics, cardiac arrest features and on-scene intervention for OHCA receiving a resuscitation attempt and not witnessed by EMS during the pre-COVID-19 and COVID-19 periods.

	Pre-COVID-19 1st January 2017-15th March 2020 N = 34,194	COVID-19 16th March 2020-31st December 2021 N = 19,854
Age, median (IQR)	65 (50, 77)	66 (50, 77)
Male, % (n)	68.7 (23,506)	68.7 (13,647)
Location, % (n)		
Private residence	71.3 (24,381)	75.3 (14,954)
Public place	20.1 (6,873)	17.4 (3,446)
Other	8.6 (2,940)	7.3 (1,454)
Bystander witnessed arrest, % (n)	52.8 (18,059)	53.4 (10,599)
Aetiology, % (n)		
Medical	86.2 (29,473)	86.3 (17,131)
Non-medical	13.8 (4,721)	13.7 (2,723)
Arrest rhythm, % (n)		
Shockable	29.2 (9,977)	26.0 (5,162)
Non-shockable	70.8 (24,217)	74.0 (14,692)
Bystander CPR, % (n)	74.7 (25,561)	76.8 (15,252)
Shocked by AED, % (n)	4.3 (1,473)	5.4 (1,081)
EMS response time (minutes), median (IQR)	8.0 (6.0, 11.0)	9.0 (6.5, 12.0)
Scene outcome, % (n)		
Died at scene	62.1 (21,243)	68.3 (13,546)
Transported	37.9 (12,946)	31.7 (6,300)
Survived event, % (n)	25.8 (8,793)	22.5 (4,460)
Survived to discharge/30 days, % (n)	11.1 (3,085)	8.9 (1,414)

### Bystander CPR

After adjustment for factors that impact bystander CPR, such as location of the arrest, there was no difference in the odds of bystander CPR across the two periods in Australia (aOR = 0.96; 95% CI:0.88–1.05, p = 0.36) or New Zealand (aOR = 0.89; 95% CI:0.74–1.08, p = 0.23) or regionally (e.g. between states in Australia) (Table 4).

**Table 4:** Trends in OHCA resuscitation attempts and trends in bystander CPR amongst OHCA receiving a resuscitation attempt.

	Bystander CPR						EMS Resuscitation attempt					
	OR	95% CI	p-value	AOR	95% CI	p-value	OR	95% CI	p-value	AOR	95% CI	p-value
Overall COVID-19	0.92	0.86–0.99	0.03	0.95	0.88–1.03	0.20	0.95	0.91–0.99	0.02	1.03	0.97–1.09	0.38
Australia COVID-19	0.93	0.86–1.01	0.10	0.96	0.88–1.05	0.36	0.96	0.91–1.00	0.08	1.02	0.96–1.09	0.51
New Zealand COVID-19	0.86	0.72–1.04	0.12	0.89	0.74–1.08	0.23	0.93	0.83–1.03	0.15	1.03	0.89–1.20	0.69
**EMS**												
VIC COVID-19	0.96	0.83–1.11	0.57	0.96	0.82–1.12	0.63	0.93	0.85–1.01	0.09	1.00	0.88–1.12	0.96
SA COVID-19	1.17	0.90–1.54	0.24	1.22	0.92–1.62	0.16	1.00	0.83–1.21	0.97	0.96	0.75–1.23	0.77
WA COVID-19	0.74	0.58–0.96	0.02	0.79	0.61–1.02	0.07	1.07	0.94–1.23	0.29	1.26	1.03–1.54	0.02
QLD COVID-19	0.83	0.69–1.00	0.05	0.87	0.72–1.05	0.15	1.04	0.92–1.17	0.56	1.19	1.01–1.40	0.03
NT COVID-19	0.87	0.37–2.04	0.75	0.73	0.30–1.77	0.48	NA	NA	NA	NA	NA	NA
NZ^1^ COVID-19	0.89	0.74–1.08	0.25	0.92	0.75–1.13	0.43	0.94	0.85–1.05	0.30	1.06	0.91–1.25	0.44
Wellington (NZ) COVID-19	0.66	0.38–1.15	0.14	0.64	0.36–1.16	0.14	1.23	0.85–1.77	0.27	1.21	0.73–2.03	0.46
TAS^2^ COVID-19	1.69	0.92–3.07	0.09	1.65	0.88–3.08	0.12	0.87	0.63–1.21	0.42	0.65	0.42–1.01	0.054
ACT COVID-19	0.29	0.08–1.02	0.054	0.34	0.08–1.38	0.13	NA	NA	NA	NA	NA	NA
NSW COVID-19	0.92	0.78–1.07	0.27	0.96	0.82–1.13	0.64	0.95	0.87–1.03	0.21	0.97	0.86–1.09	0.62

OR: Crude odds ratio; AOR: Adjusted odds ratio.

Reference category is the pre-COVID-19 period (Jan 2017 to March 2020).

95% CI: 95% confidence interval.

AOR adjusted for patient age and sex, arrest location, arrest aetiology, initial arrest rhythm (for EMS resuscitation attempt only), witnessed status, bystander CPR (for EMS resuscitation attempt only), EMS response times, season and annual trend.

NA: data not available.

VIC: Victoria; SA: South Australia; WA: Western Australia; QLD: Queensland; NT: Northern Territory; NZ: New Zealand; TAS: Tasmania; ACT: Australian Capital Territory; NSW: New South Wales.

1 Includes New Zealand except for the Greater Wellington region

2 Industrial action in TAS during 2018 impacted on data concerning bystander CPR for that period. The models for resuscitation attempted OHCA and survival outcomes were not adjusted for bystander CPR and the analysis of bystander CPR excluded the 2018 data.

### EMS resuscitation attempts

The adjusted odds ratios indicate that the COVID-19 pandemic was not associated with EMS-attempted resuscitation overall, or at the national level in Australia and New Zealand (Table 4). With respect to individual regions, COVID-19 was only associated with a significant increase in the odds of a resuscitation attempt in Western Australia (aOR = 1.26; 95% CI:1.03–1.54, p = 0.02) and Queensland (aOR = 1.19; 95% CI:1.01–1.40, p = 0.03) (Table 4).

### OHCA survival

The COVID-19 period was associated with significantly lower odds of event survival in Australia (aOR: 0.91; 95% CI:0.83–0.99, p = 0.04), and within Australia, in Victoria (aOR = 0.74; 95% CI:0.63–0.87, p < 0.001) and Tasmania (aOR = 0.48; 95% CI:0.25–0.91, p = 0.02) (Table 5). There were no significant differences between the pre-COVID-19 and COVID-19 periods in New Zealand (Table 5).

**Table 5:** Trends in OHCA survival outcomes amongst OHCA receiving a resuscitation attempt.

	Event survival						Survival to hospital discharge/30 days					
	OR	95% CI	p-value	AOR	95% CI	p-value	OR	95% CI	p-value	AOR	95% CI	p-value
Overall COVID-19	0.86	0.80–0.93	<0.001	0.93	0.86–1.01	0.10	0.77	0.69–0.87	<0.001	0.88	0.77–1.01	0.06
Australia COVID-19	0.84	0.78–0.91	<0.001	0.91	0.83–0.99	0.04	0.73	0.64–0.83	<0.001	0.82	0.70–0.96	0.01
New Zealand COVID-19	0.95	0.80–1.14	0.61	1.05	0.86–1.27	0.64	0.92	0.73–1.15	0.45	1.07	0.82–1.39	0.61
**EMS**												
VIC COVID-19	0.72	0.62–0.83	<0.001	0.74	0.63–0.87	<0.01	0.68	0.54–0.84	<0.001	0.70	0.54–0.91	0.008
SA COVID-19	0.89	0.67–1.19	0.44	1.01	0.73–1.39	0.96	0.52	0.34–0.80	0.003	0.61	0.37–0.99	0.04
WA COVID-19	1.09	0.83–1.43	0.53	1.23	0.91–1.66	0.18	0.74	0.52–1.07	0.11	0.84	0.54–1.29	0.43
QAS COVID-19	0.95	0.79–1.14	0.59	1.03	0.85–1.25	0.79	NA	NA	NA	NA	NA	NA
NT COVID-19	0.90	0.31–2.57	0.84	1.00	0.32–3.10	0.999	NA	NA	NA	NA	NA	NA
NZ^1^ COVID-19	0.92	0.77–1.11	0.40	1.02	0.83–1.25	0.86	0.95	0.74–1.21	0.66	1.14	0.86–1.50	0.36
Wellington (NZ) COVID-19	1.35	0.81–2.26	0.25	1.40	0.79–2.48	0.25	0.73	0.37–1.43	0.36	0.61	0.27–1.38	0.23
TAS^2^ COVID-19	0.46	0.26–0.83	0.01	0.48	0.25–0.91	0.02	0.65	0.28–1.50	0.32	0.88	0.32–2.47	0.81
ACT COVID-19	1.48	0.64–3.45	0.36	1.50	0.54–4.16	0.43	NA	NA	NA	NA	NA	NA
NSW COVID-19	0.85	0.73–0.99	0.04	0.93	0.79–1.10	0.39	0.86	0.69–1.07	0.19	1.00	0.77–1.29	1.00

OR: Crude odds ratio; AOR: Adjusted odds ratio.

Reference category is the pre-COVID-19 period (Jan 2017 to March 2020).

95% CI: 95% confidence interval.

AOR adjusted for patient age and sex, arrest location, arrest aetiology, initial arrest rhythm, witnessed status, bystander CPR, EMS response times, season and annual trend.

NA: data not available.

VIC: Victoria; SA: South Australia; WA: Western Australia; QLD: Queensland; NT: Northern Territory; NZ: New Zealand; TAS: Tasmania; ACT: Australian Capital Territory; NSW: New South Wales.

1 Includes New Zealand except for the Greater Wellington region

2 Industrial action in TAS during 2018 impacted on data concerning bystander CPR for that period. The models for resuscitation attempted OHCA and survival outcomes were not adjusted for bystander CPR and the analysis of bystander CPR excluded the 2018 data.

When compared to the pre-COVID-19 period, the COVID-19 period was associated with a significant decrease in the adjusted odds of survival to hospital discharge/30 days in Australia overall (aOR = 0.82; 95% CI:0.70–0.96, p = 0.01) and in Victoria (aOR = 0.70; 95% CI:0.54–0.91, p < 0.01) and South Australia (aOR = 0.61; 95% CI:0.37–0.99, p = 0.04) (Table 5). There were no significant differences in remaining regions across Australia or in New Zealand (Table 5).

## Discussion

Our study expands on regional reports from Australia during the first wave of the COVID-19 pandemic. In our larger and longer examination across all of Australia and New Zealand, we saw a slight increase in OHCA incidence, changes to the characteristics of the cases and regional variation in rates of attempted resuscitation and survival.

While previous studies in Australia only covered the first wave of the pandemic,^9^, ^10^ ours also covered subsequent waves, including the second wave from June to November 2020 and the third wave between June and November 2021. There were fundamental differences between the first and subsequent waves in the number of COVID-19 cases and case spread.^18^ In the first wave, COVID-19 cases were predominantly overseas-acquired and spread was largely managed through international border closures and mandatory two-week hotel quarantining of Australian international arrivals. The second wave was much bigger than the first and had much more community spread. However, it was largely localised to Victoria – the remaining Australian states closed their borders to Victoria which kept the rest of the nation largely COVID-19 free.^18^ The third wave resulted in the highest case counts in Australia and New Zealand, with New South Wales and Victoria hardest hit (Supplementary file^,^ Table S1).

In our study, when compared to the pre-COVID-19 period, the incidence of OHCA was higher in the COVID-19 period in all regions except South Australia and Tasmania. It is not clear why South Australia and Tasmania differed from the rest of the region. Nonetheless, the increase seen in most regions does not appear to be related to the local incidence of COVID-19. For example, the two regions with the highest incidence of COVID-19 cases had increases in OHCA incidence that were comparable to other regions with much lower COVID-19 cases. Changes in healthcare-seeking behaviour may account for some of the increased incidence of OHCA during COVID-19. Fear of contracting COVID-19 and concerns about burdening the already stressed healthcare system may have led to delays in regular medical check-ups, in medical presentations for the evaluation of pre-OHCA symptoms and in the actions taken for onset of prodromal symptoms^19^, ^20^ resulting in increases in OHCAs.

Similar to other regions,^3^, ^4^, ^9^, ^10^ the COVID-19 period also saw changes in the demographics and arrest characteristics of OHCAs in Australia and New Zealand. Factors associated with improved survival outcomes were lower, with fewer arrests occurring in public places and in an initial shockable rhythm. The proportion of arrests in the home increased during COVID-19, most likely due to the associated lockdowns and working from home orders.

Evidence concerning the impact of COVID-19 on bystander CPR and AED use is mixed with some studies reporting no differences,^5^, ^10^ and others reporting lower rates during COVID-19.^6^, ^8^ The earlier studies from our region reported different effects of the pandemic on unadjusted rates of bystander CPR for resuscitation attempted cases, with one study in a high COVID-19 incidence region reporting lower rates of bystander CPR.^3^, ^4^ Our study, with a larger population and longer duration, found no difference in rates of bystander CPR for OHCA patients attended by EMS (excluding EMS-witnessed), nor in the subgroup receiving a resuscitation attempt after adjustment for differences in patient demographics and arrest characteristics. The absence of an association between COVID-19 and bystander CPR seems counterintuitive, given that CPR increases the risk of exposure to COVID-19. However, this may reflect an attitude-behaviour gap where people are willing to administer CPR to family members despite concerns over COVID-19 transmission.^21^ Individuals are also more likely to know of a family member’s infection status and may have fewer concerns about performing compression-only CPR offered in the emergency call for adults. Unlike earlier studies,^3^, ^4^ we found AED use in our region increased slightly during the pandemic. While this finding is surprising given the decrease in OHCAs occurring in public locations, it may reflect improvements in access to public access defibrillation over the study period (e.g., alerted responder programs) rather than reflect changes related to the pandemic.

We found no significant differences in the odds of receiving an EMS resuscitation attempt (including bystander AED use with ROSC) at the national level in Australia or New Zealand. However, regional differences were observed, with the odds of a resuscitation attempt being significantly higher during COVID-19 in Western Australia and Queensland. While COVID-19 was not associated with a lower odds of an EMS resuscitation attempt in our study, EMS response times were longer in our study and others^6^, ^9^, ^10^ to screen for COVID-19 during the emergency call and to apply personal protective equipment.^9^, ^10^ Changes in on-scene treatment have also been reported in our region.^9^, ^10^ There were changes to OHCA management in some regions, including the suspension of bag-valve-mask ventilation, the early use of endotracheal tube, mechanical CPR and minimisation of airway suctioning to reduce aerosol exposure.^9^ Some of these changes may reflect the differences seen in patient outcomes.

While the balance of evidence, internationally, suggests that survival outcomes were poorer during COVID-19, systematic reviews of studies across multiple countries have found regional variation in the impact of COVID-19 on survival.^3^ Rates for event survival and survival to hospital discharge/30 days were significantly lower during COVID-19 in Australia, although this varied regionally. COVID-19 was associated with lower rates of event survival in Victoria and Tasmania and to lower rates of survival to hospital discharge/30 days in Victoria and South Australia.

Poorer survival during the pandemic has been attributed, in part, to COVID-19-related burdens on the health system.^4^ Data from Victoria have shown that the health-care system was ill-prepared to deal with a pandemic during the early stages of COVID-19 and health-care resources were concentrated on the care of patients with COVID-19.^4^ The impact of COVID-19 on health care staff numbers may also be important and may help explain some of the effect of COVID-19 on survival. For example, case numbers during the second wave of COVID-19 in Victoria did not reach a level high enough to threaten health system capacity. However, quality of care may have been impacted as 20% of the nearly 20,000 people infected during the second wave were health care workers.^18^ Roughly one-third of these were nurses and nearly half were aged-care workers.

## Limitations and strengths

Some limitations to our study should be noted. As described in the methods, some regions only collected data from OHCA cases who receive an EMS-attempted resuscitation, and not all services currently collect survival to hospital discharge or 30-days. At this time, we are unable to assess post-resuscitation care and neurological outcomes, as these data are not available in our Epistry. Finally, we did not separate the initial wave of the pandemic from later periods where case numbers and community spread were higher, however, this would have difficult in the smaller regions where both OHCA and COVID-19 cases were comparatively low. The potential for missing data bias remains a problem. In the cohort used to analysis resuscitation attempts, 18% of cases were excluded due to missing data. However, the missing data were predominantly from cases resulting in an on-scene death who had not receive a resuscitation attempt. In of this strong patterning, we viewed the missing at random assumption required for multiple imputation to be tenuous and elected to undertake a complete case analysis. Furthermore, over-adjustment bias cannot be excluded given that some of our covariates may be mediators rather than confounders. This limits our ability to draw inferences on the total effect of the pandemic on our outcomes. However, we feel that our decision to include these covariates in our models is justifiable. They were selected on the basis of earlier work where we developed a risk-adjustment algorithm to support fair, like-with-like comparisons between regions.^17^ We felt that risk adjustment was necessary as we presented results at the regional level.

Nonetheless, our study has its strengths. It is a binational, population-based study with complete or near complete capture of all OHCA attended by EMS in Australia and New Zealand. The data were collected in accordance with clearly defined and standardised guidelines and have undergone a rigorous validation process.

## Conclusion

During the COVID-19 years of our study, the incidence of OHCA increased slightly, but this change did not align the incidence of COVID-19. This period also saw changes in arrest characteristics, particularly increases in those associated with worse OHCA outcomes. While there was no change in attempted EMS resuscitation in either country, the COVID-19 period was associated with decreased survival in Australia. On further breakdown, this decrease was only seen in three regions within Australia and was sustained after adjustment for known predictors of survival. Further examination is required to understand the drivers of this change.

While resuscitation attempts increased during COVID-19 in two regions, bystander CPR rates were generally similar across the pre-COVID-19 and COVID-19 periods. Survival rates were lower during COVID-19 in Australia overall, although this only occurred in three regions after adjustment for changes in demographics and arrest characteristics. Changes in survival were likely driven by multiple factors.

## CRediT authorship contribution statement

**Stuart Howell:** Writing – original draft, Validation, Project administration, Methodology, Formal analysis, Conceptualization. **Ziad Nehme:** Writing – review & editing, Validation. **Stephen Ball:** Writing – review & editing, Validation. **Tan Doan:** Writing – review & editing, Validation. **Judith Finn:** Writing – review & editing, Validation, Funding acquisition, Conceptualization. **Emma Bosley:** Writing – review & editing, Validation. **Steven Faddy:** Writing – review & editing, Validation. **Bridget Dicker:** Writing – review & editing, Validation. **Andy Swain:** Writing – review & editing, Validation. **Peter Cameron:** Writing – review & editing, Validation. **Melanie Thorrowgood:** Writing – review & editing, Validation. **Andrew Thomas:** Writing – review & editing, Validation. **Samuel Perillo:** Writing – review & editing, Validation. **Mike McDermott:** Writing – review & editing, Validation. **Matt Green:** Writing – review & editing, Validation. **Nicole Packham:** Writing – review & editing, Validation. **Ashanti Dantanarayana:** Writing – review & editing, Validation. **Joe Cuthbertson:** Writing – review & editing, Validation. **Janet Bray:** Writing – review & editing, Validation, Supervision, Project administration, Methodology, Funding acquisition, Conceptualization.

## Funding

This study was funded by a Heart Foundation of Australia Vanguard Grant (#103010) and through a National Health and Medical Research Council (NHMRC) Centre of Research Excellence (#2035259). JB (#104751) and ZN (#105690) were funded by Heart Foundation of Australia Fellowships. JF (#1174838) was supported by NHMRC Investigator Grant.

## Declaration of competing interest

The authors declare the following financial interests/personal relationships which may be considered as potential competing interests: JB is an Editor and ZN a Editorial Board Member for Resuscitation Plus. JB and JF are Resuscitation Editorial Board Members.
