# Supplementary material for: The impact of the COVID-19 pandemic on resuscitation attempts, bystander CPR and survival outcomes in Australia and New Zealand: A binational population-based, Epistry study
Source: Resusc Plus. 2025 Feb 7;22:100894. doi: 10.1016/j.resplu.2025.100894 (PMC11880727; doi:10.1016/j.resplu.2025.100894)
Supplement: Supplementary Data 1 [file mmc1.docx]

**COVID in Australia and New Zealand (Table S1)**

In Australia, the COVID pandemic was characterised by three distinct waves during our study period (March 16, 2020 to December 31, 2021) (Table 1). The first wave encompassed the period March 16, 2020, when the federal government declared a state of emergency amid rising cases, to mid-May, 2020 when new daily cases dropped to below 20. The second wave emerged in mid-June and lasted until the end of October in 2020. The second wave was bigger than the first, had greater community spread but was largely localised to Victoria – 92.8% of cases in Australia during the second wave were recorded in Victoria. A third wave in emerged mid-June, 2021 and lasted beyond December 31 of that same year. This produced the highest COVID caseload of all three waves, accounting for 92.5% of all COVID cases in Australia during the study period (March 2020-December 2021). New South Wales and Victoria bore the brunt of the third wave, and in combination, accounted for 92.5% of cases in Australia for that period.

Over the same study period, there were two distinct waves of COVID in New Zealand, with the first between March 16 to May 17, 2020 and the second between June 1 and December 31, 2021 (Table 1). The latter wave accounted for 81.3% of COVID cases in New Zealand over the study period.

The third wave in Australia (second wave in New Zealand) coincided with the emergence of the COVID delta strain, which was more virulent than earlier COVID strains. The experience of New South Wales and Victoria indicated that the test, trace and isolate strategy was largely ineffective for managing the delta variant and that it was too infectious to be eliminated by strict lockdowns.(1, 2) Australia subsequently progressed to a policy of management through vaccination and restrictions were progressively eased as vaccination rates exceeded 70%.

Table S1 COVID case counts by wave of COVID pandemic in Australia and New Zealand

|  | First Wave: Mar 16, 2020-May 17, 2020 | | Second Wave: June 15, 2020-Oct 31, 2020 | | Nov 1, 2020-May 31, 2021 | | Third Wave: June 1, 2021-Dec 31, 2021 | | Total cases: Mar 16, 2020-Dec 31, 2021 | |
| --- | --- | --- | --- | --- | --- | --- | --- | --- | --- | --- |
|  | N cases | Percent of total^1^ | N cases | Percent of total^1^ | N cases | Percent of total^1^ | N cases | Percent of total^1^ | N cases | Percent of total |
| VIC | 1,504 | 0.9 | 18,614 | 10.5 | 252 | 0.1 | 155,936 | 88.4 | 176,477 | 100 |
| NSW | 3,130 | 1.7 | 1,101 | 0.6 | 1,166 | 0.6 | 181,917 | 97.1 | 187,370 | 100 |
| QLD | 994 | 7.2 | 106 | 0.8 | 447 | 3.2 | 12,245 | 88.7 | 13,802 | 100 |
| WA | 538 | 47.2 | 166 | 14.6 | 250 | 21.9 | 140 | 12.3 | 1,140 | 100 |
| SA | 419 | 3.8 | 61 | 0.6 | 253 | 2.3 | 10,324 | 93.4 | 11,058 | 100 |
| TAS | 220 | 28.2 | 2 | 0.3 | 4 | 0.5 | 551 | 70.7 | 779 | 100 |
| ACT | 106 | 2.6 | 6 | 0.1 | 10 | 0.2 | 3,886 | 96.9 | 4,009 | 100 |
| NT | 29 | 5.1 | 9 | 1.6 | 135 | 23.6 | 399 | 69.8 | 572 | 100 |
| Australia | 6,942 | 1.8 | 20,065 | 5.1 | 2,517 | 0.6 | 365,398 | 92.5 | 395,207 | 100 |
|  |  |  |  |  |  |  |  |  |  |  |
| New Zealand | 1,493 | 10.5 | 460 | 3.2 | 702 | 4.9 | 11,529 | 81.3 | 14,185 | 100 |

^1^Percentage of total cases between 16^th^ March, 2020 and December 31^st^, 2021

Data sources:

Australia: <https://covidlive.com.au/archive>

New Zealand: <https://github.com/minhealthnz/nz-covid-data/blob/main/cases/covid-case-counts.csv>

Table S2: Percentages for unknown response and missing data in EMS attended OHCA excluding EMS witnessed arrests

|  | All attended | | EMS resuscitation | |
| --- | --- | --- | --- | --- |
|  | Pre-COVID-19  N=91,668 | COVID-19  N=55,478 | Pre-COVID-19  N=37,083 | COVID-19  N=21,278 |
| Age, n (%)  Unknown  Missing | 67 (0.1)  1,760 (1.9) | 85 (0.1)  1,293 (2.33) | 21 (0.1)  233 (0.6) | 19 (0.1)  353 (1.7) |
| Sex, n (%)  Unknown  Missing | 402 (0.4)  43 (0.0) | 186 (0.3)  6 (0.0) | 21 (0.1)  10 (0.0) | 6 (0.0)  0 |
| Location, n (%)  Unknown  Missing | 86 (0.1)  685 (0.7) | 57 (0.1)  0 (0.0) | 44 (0.1)  120 (0.3) | 24 (0.1)  0 |
| Witnessed status, n (%)  Unknown  Missing | 3,170 (3.5%)  301 (0.3) | 1,004 (1.8)  126 (0.2) | 1,350 (3.6)  138 (0.4) | 433 (2.0)  49 (0.2) |
| Aetiology, n (%)  Unknown  Missing | 2,082 (2.3)  174 (0.2) | 1,704 (3.1)  88 (0.2) | 742 (2.0)  62 (0.2) | 601 (2.8)  29 (0.1) |
| Initial rhythm, n (%)  Unknown  Missing | 5,895 (6.4)  5,479 (6.0) | 3,297 (5.9)  3,241 (5.8) | 453 (1.2)  144 (0.4) | 376 (1.8)  33 (0.2) |
| Bystander CPR, n (%)  Unknown  Missing | 1,202 (1.3)  1 (0.0) | 469 (0.8)  2 (0.0) | 599 (1.6)  1 (0.0) | 233 (1.0)  1 (0.0) |
| AED use, n (%)  Unknown  Missing | 14 (0.0)  4 (0.0) | 0 (0.0)  5 (0.0) | 5 (0.0)  4 (0.0) | 0  5 (0.0) |
| Response time, n (%)  Unknown  Missing | 0  119 (0.1) | 0  28 (0.0) | 0  39 (0.1) | 0  10 (0.0) |
| Scene outcome, n (%)  Unknown  Missing | 1 (0.0)  32 (0.0) | 30 (0.0)  92 (0.2) | 1 (0.0)  18 (0.0) | 16 (0.1)  33 (0.2) |
| Event survival, n (%)  Unknown  Missing | 115 (0.1)  43 (0.0) | 70 (0.1)  5 (0.0) | 114 (0.3)  11 (0.0) | 52 (0.2)  5 (0.0) |
| Survival to discharge/30 days, n (%)  Unknown  Missing | 315 (0.4)  300 (0.4) | 57 (0.1)  218 (0.5) | 290 (1.0)  276 (0.9) | 52 (0.3)  207 (1.2) |

Table S3: Population estimates used for the calculation of OHCA incidence rates

|  | Land area (km^2^) | Population density (persons per km^2^) | Pre-COVID  (Population as at June 2018) | COVID  (Population as at June 2020) |
| --- | --- | --- | --- | --- |
| New South Wales | 801,150 | 10.1 | 7,954,476 | 8,110,610 |
| Victoria | 227,444 | 29.1 | 6,423,038 | 6,615046 |
| Queensland | 1,729,742 | 3.0 | 5,006,623 | 5,165,613 |
| South Australia | 984,321 | 1.8 | 1,746,137 | 1,790,355 |
| Western Australia | 2,527,013 | 1.1 | 2,617,792 | 2,712,912 |
| Tasmania | 68,401 | 8.2 | 537,291 | 557,578 |
| Northern Territory | 1,347,791 | 0.2 | 247,095 | 247,428 |
| Australian Capital Territory | 2,358 | 188.7 | 426,081 | 444,903 |
| Australia total | 7,688,220 | 3.3 | 24,963,258 | 25,649,248 |
|  |  |  |  |  |
| New Zealand (excluding Wellington region) | 260,726 | 17.4 | 4,374,700 | 4,548,100 |
| Wellington | 7,589 | 71.3 | 525,900 | 541,400 |
| New Zealand total | 268,315 | 19.0 | 4,900,600 | 5,089,500 |

Data sources:

Australian Estimated Resident Population (ERP): Australian Bureau of Statistics (ABS)

New Zealand Subnational population estimates: Stats NZ

References

1. Bennett CM. Learning to live with COVID-19 in Australia: time for a new approach. Public Health Res Pract. 2021;31(3).

2. Kenyon G. Australia's struggle with the delta variant. Lancet Infect Dis. 2021;21(10):1358.
